# Third Editor

**Published:** 1853-10

**Authors:** 


*Third Editor.*—The importance of a knowledge of chemistry to the practitioner of dental surgery, being now generally admitted, the conductors of the Journal have determined to extend the scope of their publication by introducing into it a new department—one devoted exclusively to chemistry and metallurgy, as applied to dentistry. To do this, will require a greater additional amount of labor than their arduous professional duties will permit them to bestow upon the subject. They are happy to announce, however, that they have secured the services of Dr. A. S. Piggot, a gentleman, in every respect, eminently qualified for the task, and already known to the readers of the Journal, as the author of several ably written papers on the chemistry of the fluids of the mouth, which have appeared in its pages. Dr. P. is also the author of a work just published, of about five hundred pages, on dental chemistry and metallurgy. Thus, in associating him with them in the editorship of the publication, they feel that they have been peculiarly fortunate in obtaining the assistance of a gentleman whose labors will contribute so largely to enhance the value of their work.

Having now fairly introduced Dr. Piggot to their readers, they do not deem it necessary to say more by way of commendation. They would add, however, that the Journal will continue to be conducted upon the same liberal and independent principles which have heretofore characterized its course; and, while the editors will endeavor to make it as practically useful as possible, they, at the same time, wish it to be distinctly understood, that it will not cater for any party, or clique, or set of men, but for the whole profession.

